# Beyond the Headache: Autonomic Reflex Dysfunction and Sensory Hypersensitivity Contribute to Orthostatic Intolerance in Migraine

**DOI:** 10.21203/rs.3.rs-6847469/v1

**Published:** 2025-06-16

**Authors:** Bridget R Mueller, Maya Campbell, Jihan Grant, Jasmin Jean, Marianna Vinokur, Michael Kaplan, Daniel Clauw, Jessica Robinson-Papp

**Affiliations:** Icahn School of Medicine at Mount Sinai; Icahn School of Medicine at Mount Sinai; Icahn School of Medicine at Mount Sinai; Icahn School of Medicine at Mount Sinai; Icahn School of Medicine at Mount Sinai; Icahn School of Medicine at Mount Sinai; University of Michigan; Icahn School of Medicine at Mount Sinai

**Keywords:** autonomic nervous system, cardiac reflexes, dysautonomia

## Abstract

**Objective:**

We sought to determine: 1.) the relationship between headache frequency and autonomic reflexes, and 2.) mechanisms underlying orthostatic intolerance (OI) in patients with migraine.

**Methods:**

Adults with migraine (N = 30) underwent autonomic function tests summarized as the Composite Autonomic Severity Score (CASS) and vagal/adrenergic baroreflex sensitivity (BRS-V/A). Postural Orthostatic Tachycardia Syndrome (POTS) and orthostatic hypotension/hypertension were diagnosed during tilt table testing. A cold pressor test (CPT) evaluated sympathetic vasomotor function. Participants completed the Migraine Disability Assessment (MIDAS), the 2011 Fibromyalgia (FM) Survey Criteria, chronic overlapping pain condition (COPC) screener, and Compass-31.

**Results:**

Monthly headache days correlated with CASS (p = 0.001), BRS-V (p < 0.001), and the systolic blood pressure response to CPT (p = 0.003) in the expected direction with increasing ANS reflex dysfunction correlating to increasing number of headache days. During tilt testing, OI was prevalent (25/30; 83%) and reported by all patients with chronic migraine. An abnormal cardiovascular response to tilt was present in the majority (63%) of which POTS was the most common etiology (56.2%). Patients reporting OI during tilt table testing despite a *normal* cardiovascular response (33%) had higher FM scores (15.8 ± 3.6 vs. 7.5 ± 4.6; p < 0.01) and a greater prevalence of non-headache COPCs (88.8% versus 20.0%, p = 0.02), compared to participants who were asymptomatic during tilt.

**Conclusions:**

There are two etiologies of OI in patients with migraine: 1.) an abnormal cardiovascular response to tilt (concordant OI) and, 2.) sensory hypersensitivity (discordant OI).

## 1.0 Introduction

Migraine typically begins as an episodic disorder with discrete attacks of pain and sensory sensitivity that are followed by a return to neurological baseline interictally.[1–4] However, each year, approximately 3% of patients with episodic migraine (EM) develop chronic migraine (CM), defined as 15 or more monthly headache days with 8 or more meeting criteria for migraine. [5, 6] As headache frequency increases and EM transforms into CM, patients frequently experience mood changes, continuous hypersensitivity to sensory stimuli, and orthostatic intolerance (OI) (i.e. lightheadedness/dizziness, syncope, brain fog). [7–9] Studies have demonstrated alterations in central pain processing circuits contribute to persistent sensory sensitivity present in CM and other chronic pain disorders. [10–12] However, changes in pain processing circuitry have generally not been associated with OI and the pathophysiology leading to OI in CM remains poorly understood. As a result, OI is frequently misdiagnosed as unrelated to CM, and effective treatment options for OI are limited.

It is well-established that patients with migraine, especially CM, have changes in their autonomic nervous system (ANS) during a migraine and interictally. However, studies have relied on measures of *resting* heart rate variability, an index of ANS activity linked to limbic and prefrontal cortical circuitry that is not directly involved in cardiovascular homeostasis. [13] We hypothesize that dysregulated autonomic *reflexes* may provide a physiological basis for OI in patients with a high burden of migraine. The baroreflex is a critical reflex that maintains hemodynamic stability during a postural change. During orthostasis, 500–700 ml of blood from thoracic arteries shifts to lower extremity and splanchnic capacitance vessels, reducing arterial blood pressure and triggering baroreflex-mediated cardiovagal and sympathetic actions that restore baseline BP. [14] Although dysregulated baroreflex function is associated with disorders of orthostasis, including orthostatic hypotension [15] and Postural Orthostatic Tachycardia Syndrome (POTS) [16, 17], no studies have examined the relationship between reflexes and OI in patients with CM and EM. Our study will examine autonomic reflexes in patients with EM and CM to advance our understanding of mechanisms leading to OI in patients with a high burden of migraine.

The baroreflex may have special relevance to OI in individuals with migraine. Central autonomic networks involving the hypothalamus and brain stem suppress baroreflex activity during pain or emotional stress to allow the simultaneous rise of both BP and heart rate (HR), optimizing the “fight-or-flight” response [18–21] While transient stressor-evoked suppression of baroreflex sensitivity may have adaptive physiological benefits in the short-term, prolonged or repeated episodes of pain are linked to persistent suppression and dysfunction of the baroreflex, which is associated with hemodynamic instability during orthostasis. [22] Therefore, we hypothesize that autonomic reflex dysfunction may provide a physiological basis for the higher prevalence of OI in patients with CM compared to EM.

**Figure 1:**
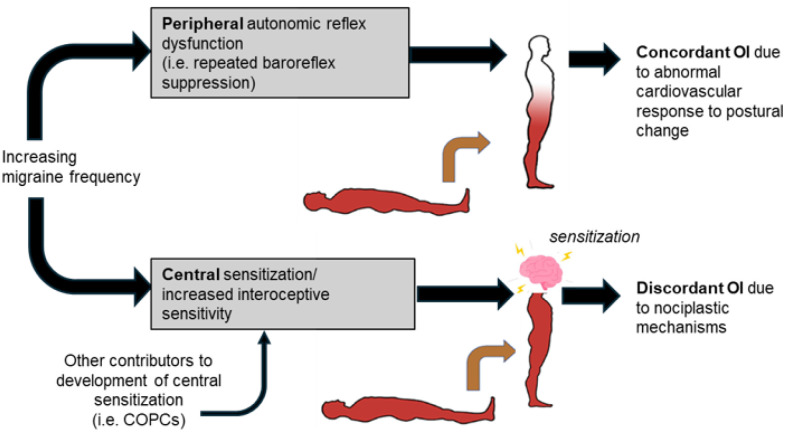
Schematic demonstrating two paths to develop orthostatic intolerance (OI) in patients with migraine.

OI, though understudied in patients with pain, has been reported in conditions including fibromyalgia [23], myalgic encephalitis/chronic fatigue syndrome (CFS) ME/CFS [24] and irritable bowel syndrome (IBS) [25–27] termed chronic overlapping pain conditions (COPCs). CM and chronic tension type headache are also among the ten recognized COPCs which arise from nociplastic pain, a distinct mechanism involving central sensitization and defined by the International Association for the Study of Pain (IASP) as *“pain that arises from altered nociception despite no clear evidence of actual or threatened tissue damage causing the activation of peripheral nociceptors or evidence for disease or lesion of the somatosensory system causing the pain.”* [28] While activation of the trigeminocervical complex results in an episode of migraine, amplification and/or dysregulation of neural circuits involved in sensory processing underlie the central sensitization that drives CM and other COPCs. [10, 29] Therefore, we hypothesize that dysregulated/heightened sensory processing (i.e. interoception) is a second important pathology, distinct from autonomic reflex dysfunction, contributing to OI in patients with a high burden of migraine, which may be referred to as ‘discordant’ OI, to indicate that a patient’s symptoms do not reflect pathology in the cardiovascular system. (Figure 1)

Our study had three objectives. First, we evaluated whether autonomic reflex function was associated with the frequency of monthly headache days during the preceding three months. We hypothesized that the repeated blunting of BRS during episodes of migraine would ultimately lead to continued suppression of BRS interictally; thus, individuals experiencing a higher number of monthly migraine days would be more likely to demonstrate reduced autonomic reflex activity compared to those with a lower frequency of monthly migraine days. Next, we evaluated the presence of OI using standard head-up tilt table testing. Given the importance of autonomic reflexes in regulating cardiovascular homeostasis during a change in body position and their reduced activity during pain, we hypothesized that patients with OI would be more likely to have both a higher frequency of monthly migraine and autonomic reflex dysfunction. Finally, we used the 2011 Fibromyalgia (FM) Survey Criteria (FM Score), COPC screener, and a multi-sensory sensitivity score (MSSS) to explore if OI correlated with validated measures of central sensitization/nociplastic pain in patients with migraine. [27, 28]

## 2.0 Materials and Methods

### 2.1 Study Design and Patient Population.

This is a cross-sectional, prospective observational study. Participants were recruited from the David S. and Ruth L. Gottesman Center for Headache Treatment and Translational Research in the Mount Sinai Health System in New York City. Eligibility criteria included a diagnosis of migraine for more than 6 months prior to autonomic testing by a headache specialist according to the international classification of headache disorders, 3^rd^ edition (ICHD-3), age between 18 and 70 years, and stable migraine treatment for at least 3 months. Patients were also required to have completed a headache diary (e-diary or paper permitted) for 3 months prior to enrollment, which is standard of care at our headache center. Both the number of days with migraine and the number of days with headache pain that did not meet ICHD-3 diagnostic criteria for migraine were recorded by patients in the diary prior to enrollment. [30] Patients with other types of headache syndromes and facial pain disorders were excluded. In addition, patients with a diagnosis known to cause autonomic dysfunction (e.g., diabetes) and those not able to complete required autonomic testing (e.g., participant must be able to stand) were excluded from participation. Participants taking daily beta blockers or stimulants were excluded, but PRN use was permitted if most recent use was > 48 hours prior to testing. All procedures were performed in accordance with a protocol approved by the Institutional Review Board of the Icahn School of Medicine at Mount Sinai (ISMMS) and all participants provided written informed consent.

### 2.2 Autonomic testing procedures:

Autonomic function tests (AFTs) are a standard battery of non-invasive tests which include sudomotor testing (QSWEAT), heart rate response to deep breathing, Valsalva maneuver (VM), and tilt table testing. Patients were tested interictally. For patients with daily or near daily headaches, a non-migraine day was chosen for testing. The QSWEAT (WRMedÒ) is performed by placing a capsule containing acetylcholine (ACh) on the skin in four standardized locations (forearm, proximal leg, distal leg, and foot). The capsule is attached to an automated system which delivers a small continuous electrical stimulus to the capsule causing iontophoresis of ACh into the skin, which triggers a reflexive sweat response collected by the capsule. The evoked sweat volume is measured and compared to standardized values. A non-invasive continuous beat-to-beat blood pressure (BP) monitoring device is attached to the participant’s finger and a 3-lead surface electrocardiogram and respiratory monitor are attached to the chest. BP, heart rate (HR), and respirations are recorded during the VM (forced exhalation to a pressure of 40 mmHg for 15 seconds), standardized paced deep breathing (HRDB), and a 10-minute head-up tilt test. During the tilt up portion of the tilt table testing, patients are asked by a trained technician to report symptoms of orthostatic intolerance (see Table 3 for complete list) every two minutes during tilt up and again after returning to supine position. [31] A symptom was included as positive only if it resolved with return to supine position.

Standard diagnostic criteria were used to identify POTS, orthostatic hypotension, and orthostatic hypertension during tilt table testing. [32] The presence of POTS *and* orthostatic hypertension supports a diagnosis of hyperadrenergic POTS; thus, if orthostatic hypertension and orthostatic tachycardia ≥ 30 bpm were both present, the patient was only counted as POTS. The change in tilt up SBP was calculated as the delta between baseline supine average in the 2 minutes prior to tilt up and the orthostatic sustained (> 30 seconds) nadir or peak SBP. For a patient with a normal cardiovascular SBP response, a representative minute was chosen without movement artifact. The change in tilt up HR was calculated as the delta between baseline supine average in the 2 minutes preceding tilt up, and the sustained tilt up (> 30 seconds) peak HR.

### 2.3 Calculation of autonomic indices:

The above-described procedures are used to calculate the Composite Autonomic Severity Score (CASS), which is an age- and sex-adjusted summary score reflecting overall autonomic function and is the sum of three sub-scores.[33] The sudomotor (i.e. peripheral, non-cardiovascular sympathetic) sub-score uses data from the QSWEAT, the parasympathetic/vagal sub-score is based on changes in HR during deep breathing and VM, and the adrenergic (i.e., cardiovascular sympathetic) sub-score is based on BP changes during VM and tilt table testing. A total CASS ≥2 is used to define autonomic reflex dysfunction (ARD).[34] Baroreflex sensitivity (BRS) was calculated as previously described.[35] Adrenergic BRS (BRS-A) is expressed in mmHg/second, and it is calculated by dividing the change in systolic blood pressure during phase 3 by the time required for SBP to recover following the release of VM.

### 2.4 Cold pressor testing procedures:

A cold pressor test (CPT) is used to evaluate an endothelial-dependent sympathetic reflex that is not under the control of the baroreflex. [36] Following a 5-minute baseline period, participants submerge their left hand to the wrist in an ice water bath for a maximum of 3 minutes while BP and HR are continuously monitored. [36] Participants rate the discomfort of the ice water bath every 30 seconds using a numeric pain rating scale (NPRS) from 0 to 10. Submerged time in ice water bath is recorded.[37] The CPT response is assessed by calculating the difference between the average baseline SBP and the maximum SBP that is reached during the ice water bath hand submersion. A SBP increase of ≥15 mmHg defines a normal CPT response. [38, 39] Participants unable to tolerate ice water bath submergence for at least 30 seconds were excluded from analysis. In addition, patients with Raynaud’s disease, coronary artery disease, or coronary artery disease risk factors including hyperlipidemia were not permitted to complete this test.

### 2.5 Medical history and patient-reported outcome (PRO) measures:

Medical history and medication information were obtained through interview and review of the electronic medical record. The three-month headache diary, tracking both migraine and non-migraine headache days, was reviewed. The Migraine Disability Assessment (MIDAS) [40] and the Chronic Overlapping Pain Condition Screener (COPCS) [41] were answered by all participants. Participants also completed the Composite Autonomic Symptom Score-31 (COMPASS-31), which assesses autonomic symptoms related to orthostatic tolerance as well as vasomotor, secretomotor, bladder, and vision function [42] and the 2011 Fibromyalgia (FM) Survey Criteria (FM Score), a measure o nociplastic pain. [43, 44] The FM Score consists of two subscales: a Widespread Pain Index (WPI) that assesses pain over the previous week in various body regions using a body map (scored 0–19), and a Symptom Severity Scale (SSS) that assesses the presence and severity of common non-cephalgic manifestations of chronic migraine including fatigue, unrefreshing sleep, cognitive dysfunction, gastrointestinal pain, and depression (scored 0–12). The FM Score is computed by adding the WPI and SSS together. Finally, a multi-sensory sensitivity score (MSSS) was comprised of three binary questions on the COPCS: frequent sensitivity to sound, odors, and bright light (yes = 1; no = 0) and the COMPASS assessment of persistent feelings of fullness or bloating after a meal (1 = never, 2 = sometimes, 3 = a lot of the time). Answers to these four questions were summed to provide a total possible MSSS from 1 to 6. To account for potentially confounding influences of medications, an anticholinergic burden (ACB) medication score was calculated for all participants, as acetylcholine is a main neurotransmitter of the ANS.

### 2.6 Statistical Analyses:

Descriptive statistics including measures of center (mean and standard deviation or median and 1st-3rd quartiles) and frequencies were performed for demographic, autonomic, and questionnaire data. The average number of monthly migraine days, non-migraine days, and total headache days was calculated from the three months of migraine diary that preceded testing date.

Spearman’s rank correlation was performed to assess relationships between 1.) headache frequency and autonomic measurements, and 2.) measures of OI and sensory sensitivity/nociplastic pain. Multivariate linear regression covaried age and/or ACB score as appropriate. CASS scoring is adjusted for age; BRS is not. To account for the influence of pain on the SBP response during the CPT, peak NPRS and time submerged in ice-water during CPT were covaried in multivariate regression. The Fischer’s exact t-test or Mann-Whitney U test compared sensory sensitivity/nociplastic pain (i.e. FM score, COPCs, MSSS) between patients reporting OI and a normal cardiovascular response to tilt table testing and those without OI. All analyses were two-tailed and conducted at the alpha = 0.05 level using SPSS version 24.

## 3.0 Results

### 3.1 Study population demographics and clinical characteristics

Participants (N = 30) had an average age of 41 years (range: 23 to 67) and all but one were female (Table 1). The majority reported a long history of migraine with a median of 23 years. Our participants reported a median of 12 headache days per month [IQR: 9–24], the majority of which were due to migraine (8.2 [IQR: 4–11]) while a smaller number (4.0 [IQR: 0–10]) resulted from headaches that did not meet migraine criteria. The median MIDAS score of 30.5 [IQR: 10.5, 62.0] reflects significant migraine-related disability. One-third of our study population met criteria for CM, and approximately 40% reported migraine with aura. Most participants reported using prescription treatments for the prevention (76.7%) and acute (96.7%) treatment of migraine (**Supplemental Table 1**). The mean ± standard deviation (SD) ACB score for participants was 0.73 ± 1.048 and ranged from 0 to 4. (Table 1) Anxiety (53.3%) and depression(36.6%) were the most common medical comorbidities (**Supplemental Table 2**).

More than half of our study participants had at least one COPC that was not a primary headache disorder (54.4%) (**Supplemental Table 2**) and the mean ± SD FM score was 12.7 ± 6.5, a score suggestive of nociplastic pain (Table 1).[45] The median total COMPASS-31 score of 32.1 [20.9, 38.6] indicates clinically significant symptoms of dysautonomia, which was driven primarily by elevations in orthostatic and gastrointestinal subdomain scores. (Table 1)

### 3.2 Autonomic reflex dysfunction (ARD) was common in patients with migraine

#### Composite Autonomic Severity Score (CASS):

The total observed CASS ranged from 0 to 7 (out of a possible range of 0–10, where 10 is the most abnormal) and ARD, defined as a total CASS ≥ 2, was present in 33% of our study population. (Table 2) Approximately half of the participants exhibited adrenergic (46.7%) and/or cardiovagal (43.3%) dysfunction. All observed deficits were classified as mild to moderate in severity, as indicated by CASS sub-scores: all adrenergic deficits were scored as 1 out of a possible 4, and cardiovagal deficits ranged from 1 to 2 out of a possible 3. Approximately one quarter of individuals had abnormalities in sudomotor activity (23.3%). Again, as indicated by CASS sub-scores, the majority (71.4%) of these deficits were mild or moderate, ranging from 1 to 2 out of a possible 3.

#### Baroreflex sensitivity (BRS):

Adrenergic BRS (BRS-A) was below the normal threshold of 25 mmHg/seconds in 29 out of 30 participants. Of these, 27 exhibited mild to moderate impairment (5.0 mmHg/second), while two participants demonstrated a severe deficit in BRS-A (< 5.0 mmHg/second). Cardiovagal BRS (BRS-V) was below normal (< 4.0 milliseconds/mmHg) in 13 out of 30 participants and the majority (78%) had mild or moderate deficits (2.0–3.9 milliseconds /mmHg).

#### Cold Pressor Testing (CPT):

26 participants completed CPT testing. Two were excluded due to cardiovascular risk factors and two participants were unable to tolerate at least 30 seconds of left-hand ice water submersion. The SBP response to the CPT was below the expected rise of 15 mmHg in 12 of 26 (46%) participants and the SBP decreased below baseline in 5 participants (19.5%) (Table 2).

### 3.3 Increasing severity of ARD correlates with increasing frequency of headache days

#### Composite Autonomic Severity Score (CASS):

**Figure 2:**
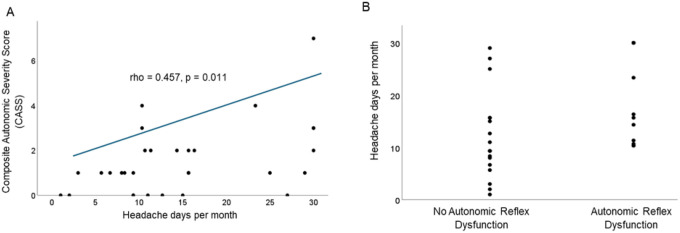
A) Positive association between total monthly headache days (average from 3 months of headache diary preceding testing) and CASS. B.) Only patients with 10 or more headache days per month satisfied criteria for autonomic reflex dysfunction (CASS ≥ 2)

Total CASS score correlated with total monthly headache days (Spearman’s rho = 0.490, p = 0.001) and the MIDAS (Spearman’s rho = 0.578, p < 0.001) in the hypothesized direction (Figure 2A). Only individuals with an average of 10 or more headache days per month had ARD (Figure 2B). In multivariate regression, CASS maintained its significant association with monthly headache days (p = 0.012) and MIDAS score (p = 0.001). We next used regression analysis to determine if the association between CASS and monthly headache days was driven by the frequency of migraine days, non-migraine days or both. We found that the relationship between CASS and monthly headache days was driven by the number of monthly non-migraine headache days (p = 0.024) only. There was no association between CASS and migraine days (p = 0.496).

#### Baroreflex sensitivity (BRS):

**Figure 3:**
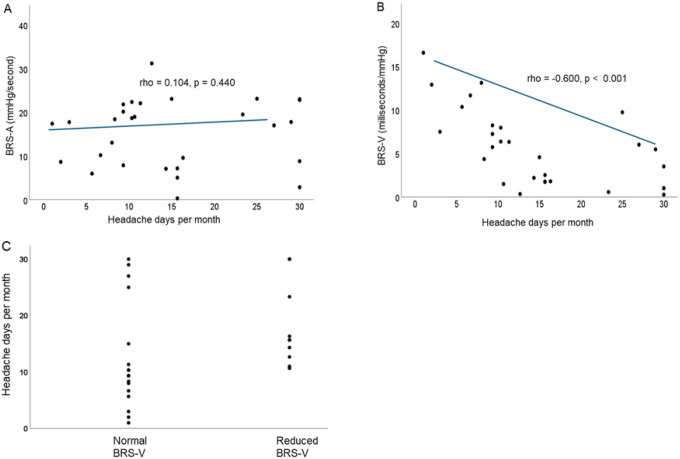
A) There is no relationship between total monthly headache days and adrenergic baroreflex sensitivity (BRS-A). All but one patient was below the normal cut-off of 25 mmHg/second. B.) Lower vagal baroreflex sensitivity (BRS-V) is associated with higher average headache days per month (average from 3 months of headache diary preceding testing). C.) Only patients with 10 or more headache days per month had vagal baroreflex sensitivity (BRS-V) below the normal threshold of 4.0 milliseconds/mmHg.

While there was no relationship between BRS-A and total monthly headache days (Figure 3A), BRS-V negatively correlated to total headache days in the expected direction (Spearman’s rho = −0.600, p < 0.001,) with an increase in headache day frequency correlating to lower BRS-V (Figure 3B). The relationship between BRS-V and total headache days maintained its association in multivariate regression (p < 0.001) and only participants with an average of 10 or more headache days per month had reduced BRS-V (Figure 3C). We next determined if the relationship between BRS-V and monthly headache days was driven by the frequency of migraine days, non-migraine days or both. We found that BRS-V was associated with the number of non-migraine days (p = 0.021), but *not* migraine days (p = 0.065).

#### Cold Pressor Testing (CPT):

**Figure 4:**
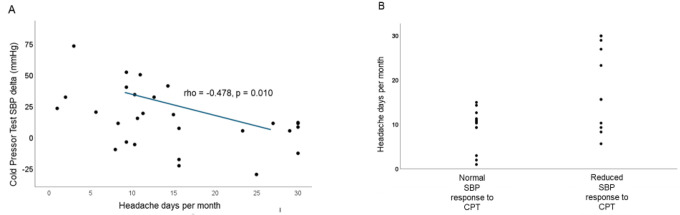
A) A lower systolic blood pressure (SBP) response to cold pressor testing (CPT) is associated with higher average headache days per month (average is obtained from 3 months of headache diary preceding testing). B.) Reduced SBP response to CPT (defined as < 15 mmHg during the first minute of left-hand immersion in ice water) is present in patients with low frequency episodic migraine, high frequency episodic migraine, and those with chronic migraine. Abbreviations not previously defined: mmHg: millimeters of mercury.

In CPT, increasing monthly headache days negatively correlated to the SBP response to left-hand ice water submersion (Spearman’s rho = −0.555; p = 0.003; Figure 4A). This relationship persisted in multivariate regression corrected for age, ice water submersion time, and maximum NPRS (p = 0.015). We examined if the relationship between SBP response during the CPT was driven by migraine days or non-migraine days and again found a significant correlation only with the number of *non*-migraine headache days (Spearman’s rho = 0.412, p = 0.024); however, this association did not maintain significance in multivariate analysis (p = 0.08). All patients with CM exhibited an abnormally reduced SBP response to the CPT; however, a reduced response was also observed in several individuals with low-frequency episodic migraine. (Figure 4B)

#### Tilt table testing:

The number of monthly headache days negatively correlated with SBP change during 10-minute tilt up challenge (Spearman’s rho = −0.415, p = 0.023) and positively correlated to HR change (rho = 0.391, p = 0.033). Thus, patients with a higher frequency of monthly headaches were more likely to show a decrease in orthostatic SBP and an increase in orthostatic HR.

### 3.4 Orthostatic intolerance (OI) was associated with chronic migraine (CM) and an abnormal cardiovascular response to tilt in two-thirds of participants (concordant OI)

**Figure 5:**
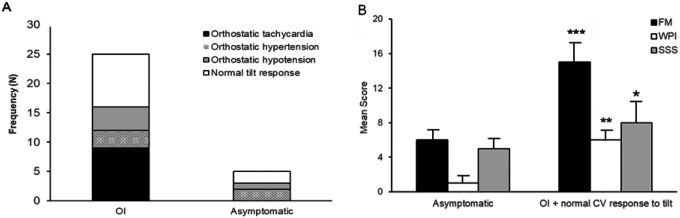
A) Cardiovascular (CV) phenotypes of participants reporting orthostatic intolerance (OI) during tilt table testing patients who remained asymptomatic. B.) Patients with OI and a normal CV response to tilt up position had an elevated 2011 Fibromyalgia (FM) Survey Criteria (FM Score) compared to patients who were asymptomatic during tilt table testing. Both components of the FM Score, the widespread pain index (WPI) and sensory sensitivity scale (SSS) were also significantly elevated when compared to asymptomatic patients. ***, p = 0.001; **, p = 0.005; *, p = 0.032

Most participants (25/30; 83.3%) reported at least one symptom of OI during tilt table testing (Table 3, Figure 5A). The three most common symptoms reported were dizziness or lightheadedness (46.7%), new or worsening headache (40.0%), and nausea (30.0%). OI associated with a diagnosis of CM (p = 0.03), all patients with CM reported OI. There was a significant correlation between the number of OI symptoms during tilt table testing and measures of nociplastic pain including number of non-headache COPCs (Spearman’s rho = 0.331, p = 0.029), FM score (Spearman’s rho = 0.326, p = 0.039), and MSSS (Spearman’s rho = 0.320, p = 0.033) but not headache days (p = 0.451) Approximately two-thirds (64%) of participants with OI had an abnormal cardiovascular response to tilt that fulfilled diagnostic criteria for one of the three orthostatic phenotypes described below:

#### POTS:

36% of participants with OI met criteria for POTS. The majority of participants with POTS had a transient decrease in SBP following tilt up, indicating orthostatic tachycardia was reflexive. Participants with POTS also had a reduced SBP response during the CPT compared to those with a normal cardiovascular response to tilt (8.2 mmHg versus 30.5 mmHg; p = 0.04). A greater proportion of patients with POTS met criteria for CM compared to those with a normal cardiovascular response to tilt (66.3% versus 33.3%; p = 0.028)

#### Orthostatic hypotension:

16% of participants with OI met criteria for orthostatic hypotension; BRS-V was significantly lower in patients with orthostatic hypotension compared to those with a normal cardiovascular response to tilt (1.1 ± 0.8 versus 7.56 ± 5.4; p = 0.021).

#### Orthostatic hypertension:

12% of participants with OI met criteria for orthostatic hypertension. No abnormal reflexes were detected in patients with orthostatic hypertension.

### 3.5 Orthostatic intolerance (OI) was associated with a normal cardiovascular response to tilt and hyperresponsivity to sensory stimuli in one-third of participants (discordant OI)

One-third of participants (9/25) reported OI during tilt table testing and demonstrated a normal cardiovascular response to tilt up position. Patients with OI and a normal cardiovascular response to tilt reported a greater number of monthly headache days compared to participants without OI (14.1 ± 9.9 versus 8.7 ± 4.9); however, this difference was not statistically significant (p = 0.09). Participants with OI and a normal cardiovascular response to tilt were more likely to have a non-headache COPC (88.8% versus 20.0%, p = 0.02), a higher FM score (15.8 ± 3.6 vs. 7.5 ± 4.6; p = 0.001), and a higher multi-sensory sensitivity score (MSSS) compared to participants without OI (4.4 ± 1.1 versus 1.13 ± 2.5; p = 0.008, Figure 5B).

## 4.0 Discussion

In this study, we investigated two hypotheses: 1.) Increasing migraine frequency will correlate with autonomic reflex dysfunction (ARD), and 2.) For individuals with migraine, OI may be explained by ARD and an abnormal cardiovascular response to orthostasis (i.e. concordant OI) or sensory sensitization (i.e. discordant OI). Supporting our first hypothesis, we found increasing total headache days predicted ARD. Interestingly, we found that autonomic reflex dysfunction was predictive of the number of monthly *non*-migraine headache days, but not migraine days. Regarding our second hypothesis, we found that for the majority of individuals, OI has a plausible cardiovascular cause (i.e. POTS, orthostatic hypotension). However, for participants with a normal cardiovascular response to orthostasis, OI may result from hypersensitivity to sensory input, which is a characteristic feature of nociplastic pain syndromes.

As hypothesized, our results demonstrate a positive correlation between the severity of ARD and both the total number of monthly headache days and the level of migraine-related disability. These novel results advance our understanding of pathophysiology that explains the higher prevalence of OI in patients with a high burden of migraine compared to those with infrequent episodic migraine. [9] Only a few studies have examined autonomic reflexes in people with migraine, and they have not yielded consistent results. For example, Sanya and colleagues found cardiovagal baroreflex sensitivity (BRS) to be significantly reduced in a group of individuals with migraine that included both EM and CM compared to healthy controls. [46] However, a study by Cortelli found no difference in BRS between patients with low frequency EM and healthy controls. [47] Our findings suggest that differences in the headache frequency of the study population may account for inconsistencies in the literature examining autonomic reflexes in individuals with migraine.

We found that the relationship between ARD and total headache days was driven by the number of non-migraine days, not migraine days. Although this result was unexpected, it aligns with well-established ANS physiology and headache pathophysiology. The baroreflex is suppressed by pain, not just migraine. [18, 48] As migraines increase in frequency, and central sensitization develops, classic migraine features including unilateral severe pain may be replaced by diffuse cephalgia, a pain pattern associated with non-migraine headache. [59, 60] Therefore, patients with a sufficient number of monthly headache days (10 or more) to cause persistent BRS-V suppression are more likely to report both non-migraine and migraine headache days. An alternative (and not mutually exclusive possibility) is that dysregulated autonomic reflexes *cause* central sensitization and the resulting non-migraine headaches. Reduced BRS-V is associated with cerebral hypoperfusion, [52] gliosis [53], neuroinflammation and decreased activity of descending anti-nociceptive pathways [56–58], which are pathologies known to drive the development of central sensitization. Longitudinal studies assessing the impact of ARD correction on headache frequency and type are needed to investigate these hypotheses.

ARD, as assessed by total CASS scores and cardiovagal baroreflex sensitivity, was present only in patients experiencing an average of 10 or more headache days per month. These findings suggest that, for certain individuals, the underlying mechanisms of high-frequency episodic migraine (EM) overlaps with those of CM and supports a growing body of evidence indicating that the threshold of 15 headache days per month commonly used to differentiate EM from CM may not accurately represent patients’ treatment needs or the true burden of disease. [49] For example, a large survey study found that pain and disability levels reported by patients with high-frequency EM (10–14 headache days per month) were not significantly different from those reported by patients with CM. [49] Our results indicate that the physiological changes that underlie CM may already be present in patients with high frequency EM and future studies should investigate the utility of using autonomic reflex activity as a biomarker for impending pain chronification.

In our study, continuous beat-to-beat blood pressure monitoring enabled us to observe that, in individuals with migraine and POTS, excessive orthostatic tachycardia frequently occurred following the normal transient decrease in blood pressure following tilt up. In response to the orthostatic shift of blood from central arteries to lower extremities and the resulting 5–15 mmHg decrease in arterial SBP, baroreflex mediated norepinephrine is released from peripheral sympathetic efferents and binds to α−1 adrenergic receptors on vascular smooth muscle, leading to vasoconstriction and an increase in systemic vascular resistance. [50] Concurrently, cardiovagal input to the heart is reduced, which results in a modest (10–20 bpm), elevation in heart rate and increased cardiac output. However, for patients with POTS, the increase in heart rate exceeds 30 bpm and is persistent. POTS is a phenotype and numerous mechanisms including a hyperadrenergic state, neuropathy, and hypovolemia, may underlie its presentation. [51, 52] We found patients with migraine and POTS have an abnormally reduced SBP response during the CPT, indicating deficits in endothelial-dependent sympathetic vasoconstriction. Thus, in the setting of failed peripheral vasoconstriction, compensatory tachycardia must be persistent and robust to correct standing BP. [39] Supporting this interpretation is our result showing a strong negative correlation between orthostatic heart rate and postural SBP. These findings support previous studies that have demonstrated patients with migraine and other chronic pain conditions have widespread deficits in the sympathetic nervous system that impact vasomotor reactivity.[53] Therefore, treatment strategies that address deficits in adrenergic mediated vasomotor reactivity (e.g. alpha agonists) could be considered for the treatment of POTS in patients with comorbid migraine.

Our findings indicate that a significant proportion of individuals reporting OI during tilt table testing display a normal cardiovascular response, suggesting that OI may result from multiple mechanisms. It is well-documented that patients with migraine are hypersensitive to *external* stimuli, including light, noise, and odors. [54] [55] The increased prevalence of non-headache COPCs and elevated FM and MSSS scores in participants with OI and a normal cardiovascular response to tilt table testing raises the interesting possibility that OI may reflect heightened sensitivity to internal sensation (i.e. interoception). This hypothesis is strengthened by our results showing significant positive correlations between measures of sensory sensitivity/nociplastic pain and OI severity in all participants. Notably, all patients reporting OI despite a normal cardiovascular response had CM or a recent history of CM, aligning with studies demonstrating interoception dysfunction is frequently present in chronic pain [56, 57] and may be involved in pain chronification. [58] Interoceptive sensitivity has not been examined in patients with migraine and our results provide a rationale for future studies that determine if interoceptive sensitivity may contribute to non-cephalgic symptoms, including gastrointestinal discomfort, tinnitus, and cognitive complaints prevalent in patients with CM.

Our study has several limitations. Its cross-sectional design prohibits causal inferences, though longitudinal studies on autonomic reflexes are in progress. We selected a modest sample size due to the demands of deep phenotyping on participants and the robust associations between autonomic reflex dysfunction and headache frequency observed in our prior retrospective chart review. [21] However, replication in diverse patient populations is necessary to confirm the generalizability of findings. We did not measure plasma catecholamine levels during standing, because pain from intravenous catheter placement during tilt table testing could influence baroreflex sensitivity. However, our results justify future research directly assessing catecholamine levels in migraine patients during orthostatic challenges.

In conclusion, this study advances our understanding of the complex interplay between chronic migraine, orthostatic intolerance, and autonomic reflex dysfunction, highlighting their shared pathophysiological mechanisms. Our results reveal that autonomic dysfunction, characterized by reduced cardiovagal baroreflex sensitivity and impaired adrenergic-mediated vasoconstriction, is strongly associated with increased headache frequency and disability. Additionally, we identify sensory hyperresponsiveness as a novel mechanism contributing to OI. Future research should focus on longitudinal studies that determine if decreased headache frequency improves autonomic reflex function, and if therapeutic strategies targeting dysregulated autonomic reflexes and sensory sensitization alleviates the burden of OI.

## Supplementary Material

Supplementary Files

This is a list of supplementary files associated with this preprint. Click to download.

- SupplementalTables.docx

## Figures and Tables

**Table 1:** Patient Characteristics

Age at time of testing (mean [SD])	40.8 [12.40]
Female (N, % total)	29 (96.67%)
Duration of headache syndrome (years) (mean [SD])	23.5 [13.46]
Anticholinergic burden (ACB) score (mean [SD])	0.73 [1.0]
Migraine with aura	13 (43.3%)
Monthly headache days^a^	12.0 [9,23.8]
Monthly migraine headache days	8.1 [4.3,11.4]
Monthly nonmigraine headache days	4.0 [0, 10.1]
Migraine Disability Assessment (MIDAS)	30.5 [10.5, 62.0]
COMPASS-31 total score	32.1 [20.9, 38.6]
COMPASS-31 orthostatic intolerance sub-score	16.0 [0.0, 21.0]
COMPASS-31 vasomotor sub-score	0.0 [0.0, 0.0]
COMPASS-31 secretomotor sub-score	4.3 [0, 7.0]
COMPASS-31 gastrointestinal sub-score	8.5 [5.1, 11.4]
COMPASS-31 bladder sub-score	0.0 [0, 1.1.0]
COMPASS-31 pupillomotor sub-score	2.5 [1.7, 3.0]
2011 Survey Criteria for Fibromyalgia Survey Criteria (FM score)	12.7 ± 6.5
Widespread Pain Index (WPI)	5.9 ± 0.9
Symptom Severity Index (SSS)	6.8 ± 0.4

* Unless otherwise noted, data is median [1q, 3q]

a, 3 months headache diary prior to enrollment

**Table 2:** Autonomic Reflex Measurements

Total Composite Autonomic Severity Scale (CASS) (N = 30)	1.0 [0.8, 2.3]
Sudomotor CASS sub-score	0.0 [0.0, 1.0]
Adrenergic CASS sub-score	0.5 [0.0, 1.0]
Cardiovagal CASS sub-score	0.0 [0.0, 1.0]
BRS-V, milliseconds/mmHg (N=29)^b^	5.7 [1.8, 9.0]
BRS-A, mmHg/second (N=29)^b^	17.7 [8.2, 21.9]
**Tilt table measurements (N = 30)**	
Baseline supine SBP (mmHg)	114.0 [101.3, 121.0]
Maximum SBP during tilt up (mmHg)	123.0 [114.0, 138.3]
Minimum SBP during tilt up (mmHg)	100.0 [85.0, 111.3]
Baseline supine DBP (mmHg)	65.5 [59.0, 75.3]
Maximum DBP during tilt up (mmHg)	87.5 [81.0, 100.5]
Minimum DBP during tilt up (mmHg)	65.5 [55.0, 71.3]
Baseline supine HR (bpm)	64.2 [58.4, 74.0]
Maximum HR during tilt up (bpm)	101.7 [88.9, 123.0]
**Cold Pressor Testing (CPT) (N = 26)** ^c^	
Baseline SBP (mmHg)	117.0 [103.0, 127.5]
Maximum SBP (mmHg)	132.0 [118.0, 144.0]
Baseline DBP (mmHg)	76.0 [66.0, 82.0]
Maximum DBP (mmHg)	87.0 [71.0, 99.0]
Baseline HR (bpm)	77.0 [67.0, 85.5]
Maximum HR (bpm)	89.0 [77.0, 96.5]

a Unless otherwise noted, data is median [1q, 3q].

b One participant had a baroreflex sensitivity that could not be measured due to a minimal change in blood pressure during the Valsalva maneuver forced expiration.

c One participant was excluded from doing the CPT due to a history of Raynaud’s syndrome, one was excluded due to cardiovascular risk factors, and two individuals could not tolerate 30 seconds of left-hand ice water submersion.

Abbreviations: BRS-A: adrenergic baroreflex sensitivity; BRS-V: vagal baroreflex sensitivity; ms: milliseconds; bpm: beats per minute, SBP: systolic blood pressure; DBP: diastolic blood pressure; HR: heart rate

**Table 3:** Orthostatic Symptoms Reported During Tilt Testing

Report of ≥ 1 symptom	25 (83.3%)
Mean ± standard deviation symptoms reported	1.8 ± 1.3
Dizzy/Lightheaded	14 (46.7%)
Headache	12 (40.0%)
Nausea	9 (30.0%)
Weak/Heavy Limbs	7 (23.3%)
Shortness of breath	3 (10.0%)
Numbness/pain in extremities	4 (13.3%)
Vision changes	3 (10.0%)
Overheated	2 (6.7%)
Chest tightness/palpitations	1 (3.3%)
Brain fog	1 (3.3%)
Participant requested to end test early due to symptoms	3 (10%)

* Unless otherwise noted, data is N (% total).
